# A Review of Oil Spill Remote Sensing

**DOI:** 10.3390/s18010091

**Published:** 2017-12-30

**Authors:** Merv Fingas, Carl E. Brown

**Affiliations:** 1 Spill Science, Edmonton, AB T6W 1J6, Canada; 2 Emergencies Science and Technology Section Environment and Climate Change Canada, Gatineau, QC K1A 0H3, Canada; carl.brown@canada.ca

**Keywords:** oil spill remote sensing, oil spill detection, oil remote sensing

## Abstract

The technical aspects of oil spill remote sensing are examined and the practical uses and drawbacks of each technology are given with a focus on unfolding technology. The use of visible techniques is ubiquitous, but limited to certain observational conditions and simple applications. Infrared cameras offer some potential as oil spill sensors but have several limitations. Both techniques, although limited in capability, are widely used because of their increasing economy. The laser fluorosensor uniquely detects oil on substrates that include shoreline, water, soil, plants, ice, and snow. New commercial units have come out in the last few years. Radar detects calm areas on water and thus oil on water, because oil will reduce capillary waves on a water surface given moderate winds. Radar provides a unique option for wide area surveillance, all day or night and rainy/cloudy weather. Satellite-carried radars with their frequent overpass and high spatial resolution make these day–night and all-weather sensors essential for delineating both large spills and monitoring ship and platform oil discharges. Most strategic oil spill mapping is now being carried out using radar. Slick thickness measurements have been sought for many years. The operative technique at this time is the passive microwave. New techniques for calibration and verification have made these instruments more reliable.

## 1. Introduction

Oil spill response now includes remote sensing as an essential component. The public expectation is that oil spill extent and location are precisely mapped [1]. Response personnel can use this location information to implement countermeasures to minimize the effect of pollution. Remote sensing is used to check for illegal discharges from ships. This is important, in view of unacceptable seabird mortality resulting from illegal discharges [2].

The end use of remote sensing data is an important consideration and depends on the oil spill remote sensing equipment utilized. For a given function, several types of systems may be needed. The data end use, such as spill location, enforcement or cleanup support, may require a specific resolution or other characteristics of the data.

There are general uses of oil spill sensed data:

1. oil spill mapping for both tactical and strategic countermeasures;
2. slick detection and surveillance;
3. gathering of legal evidence;
4. law enforcement such as regarding ship discharge;
5. direct support for oil spill countermeasures;
6. slick trajectory determination.

This review will review the information on oil spill remote sensing and show the areas of improvement and areas where further development is needed.

## 2. Detection and Mapping of Oil on a Water Surface

The detection and mapping of oil on a water surface is the most common uses of oil spill remote sensing. This is carried out using either active of passive means.

### 2.1. Passive Means of Detection and Mapping

The most common means for oil spill remote sensing is to use passive observation of the sea surface to detect and map oil spills. These include the techniques of using cameras in the visible and infrared spectra. Other wavelengths such as ultraviolet and near infrared are less frequently used.

### 2.2. Use of Optical Techniques

Oil shows only minor optical properties in the region from the ultraviolet to near infrared. These are differential reflectance, and absorbance between oil and water in the given spectral region. These differential factors vary with oil type, degree of oil evaporation, weather conditions (including air moisture content), and sun illumination. Detailed oil/water spectral differentials have not been determined across the entire visible spectrum [3]. One could not categorically identify oil using only optical data in the visible region, especially if the location of the oil spill were unknown.

### 2.3. Use of the Visible Spectrum

The visible portion of the electromagnetic spectrum ranges from 400 to 700 nm. Overall, oil displays a moderately larger reflectance than water, but does not show specific absorption or reflection tendencies. Thin oil layers or sheen appear silvery to the human eye and reflect light over a wide spectral range—as far as the blue. Thick oil layers appear to be the same color as bulk oil, typically brown or black. Overall, oil has no specific spectral information that differ from the water upon which the oil floats [4]. As a consequence, processes that inspect particular spectral regions do not enhance discrimination [4]. One method of using visible spectra is to use a push-broom scanner that uses a CCD detector and an optical system to direct ground elements to different parts of the CCD detector [5]. The signal resulting from such a CCD scanner can then be processed or enhanced to yield the desired data.

Oil on water has a polarizing effect on light, so viewing oiled water with polarized lenses can improve contrast and thus oil detection [6,7]. Light reflected from a water surface reflects at 53 degrees (Brewster angle), so one can improve contrast by setting the detector at this Brewster angle (53 degrees from the vertical) [8]. This light directly reflected from the surface contains the most information on surface oil. This technique may increase contrast as much as 100% [8].

Three major obstacles interfere with the use of visible light, darkness, cloud cover, and sun glitter. Sun glitter, which is often confused for oil sheens, is problematic in visible remote sensing. Sun glitter can be reduced through signal processing techniques [9,10].

Video cameras are used extensively for oil spill monitoring. Modifications to standard cameras have improved images somewhat, however have not vastly improved oil detection capability. Light-enhanced video cameras function in complete darkness. Night-vision camera tests showed that light-enhancement can provide good imagery in total darkness [11].

Hyperspectral imaging is a technique of using a spectrometer to collect many images of the same target at different wavelengths [3]. Hyperspectral images involve extensive data and require highly advanced processing to produce an image, so they are not useful for real-time monitoring. A technique called spectral un-mixing is commonly used for characterizing individual pixels. This technique becomes important because the spatial resolution of hyperspectral sensors may be as much as several meters and different types of target materials may be recorded in each measured pixel. Hyperspectral sensing is being increasingly employed for general environmental purposes, but application to oil spills remains low [3].

**Figure 1:**
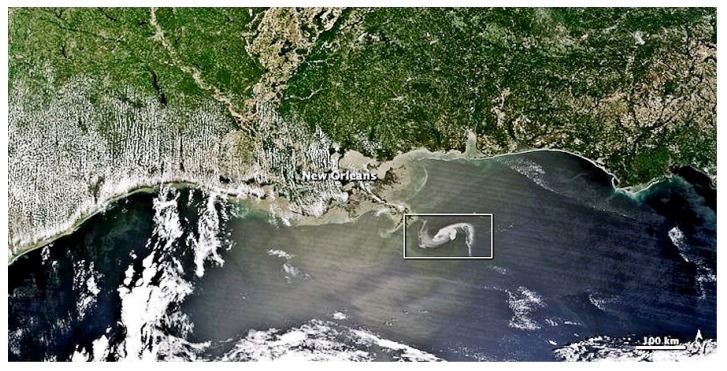
An example image from MODIS during the DWH (Deepwater Horizon) spill in the U.S. Gulf of Mexico. The MODIS sensor is on the NASA Aqua satellite. The oil slick is shown by the white rectangle. Note the many clouds in the image. (Photo from NASA website, www.nasa.gov).

**Figure 2:**
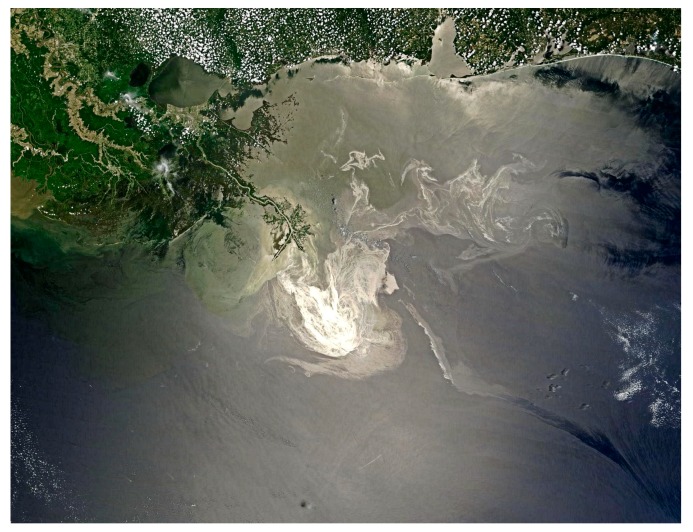
A visible image of the DWH spill. The image is from the NASA Terra satellite and was taken on 24 May 2010. Clouds do not obscure the main part of this image but are present around the periphery (photo from https://en.wikipedia.org/wiki/File:Deepwater_Horizon_oil_spill).

In summary, the visible spectrum remains a research area as well as an economical method for monitoring oil spills, particularly on countermeasures support operations. Figure 1 and Figure 2 show examples of a visible image from the Deepwater Horizon oil spill (USA Gulf of Mexico).

### 2.4. Use of the Infrared (IR)

Oil of thicknesses greater than about 10 μm, absorbs light in the visible region and re-radiates a portion of this in the infrared spectrum, mostly in the 8–14 μm wavelengths [12,13,14]. Solar-heated oil will emit infrared radiation as oil shows greater infrared emissivity than water. Thick oil appears heated or “hot” compared to the surrounding water in infrared images, intermediate thickness layers of oil appear “cool”, and thinner layers of oil or sheens are not distinguished. The transition thicknesses are not known, but indications are that the difference between the “cold” and “hot” layers lies between 50 and 150 μm and the least detectable thickness is in the range of 10–70 μm [3]. The reason for the “cool” slick occurrence may be that a 20–50 μm layer of oil on the water causes destructive interference of the IR wave fronts, thereby reducing the thermal radiation emitted. This destructive interference was shown to explain the “rainbow sheen” in the visible [3]. The cool slick then implies thicknesses between about 16 and 40 μm, because the minimum thickness resulting in destructive interference is 2 times the wavelength that is between 8 and 10 μm. This results in a destructive interference onset from 16–20 μm to 4 wavelengths or 32–40 μm. The “cool” portion is observed only on fresh or test spills, because only early phases of oil spreading yield the correct thickness to exhibit the wavelength destruction. Slicks that have resided on the sea for longer times are thicker or thinner than 16–40 μm. The threshold of the “hot” phenomenon is at thicknesses of about 50 μm that is greater than shown by the destructive layer of 16–40 μm [3].

**Figure 3:**
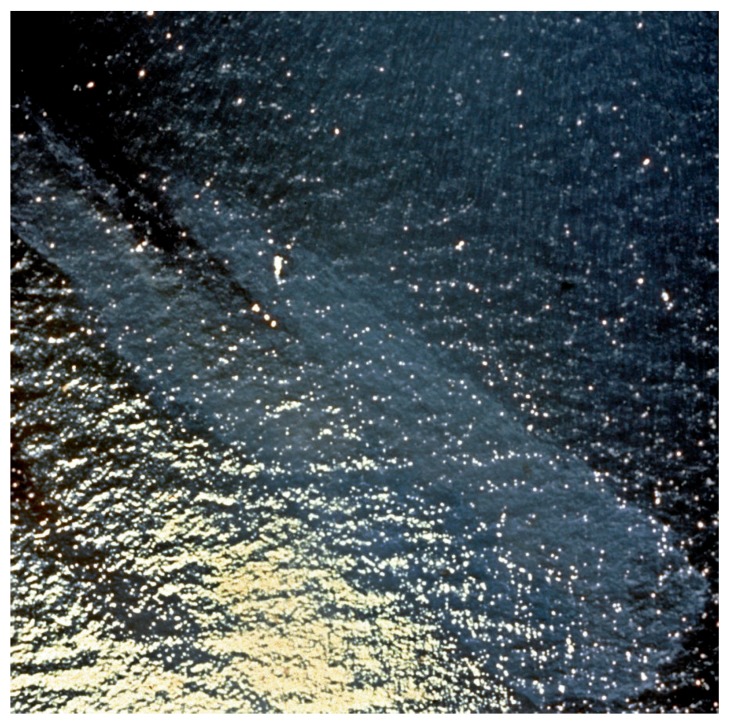
A visible photo of a small test slick. The sun glint on the left confounds the interpretation of slick edges (photo from Environment Canada).

**Figure 4:**
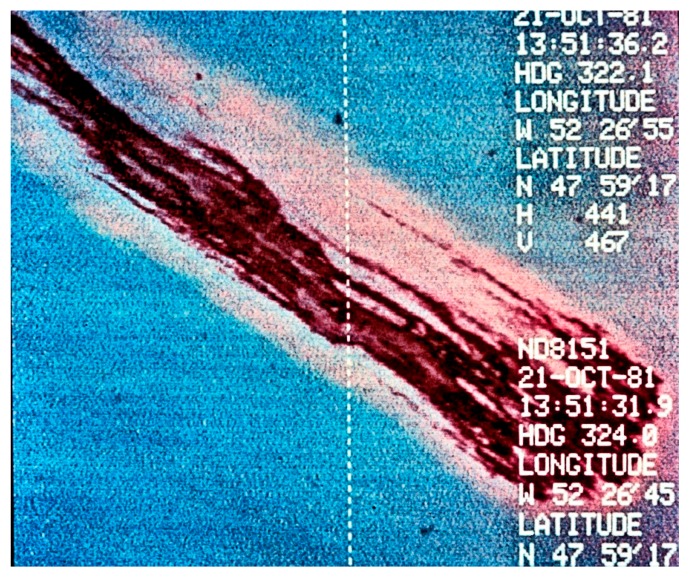
A simultaneous view of a test slick (shown in Figure 3), but using IR. The inclusion of infrared creates additional contrast between the slick and water as well as removing the sun glitter (Photo from Environment Canada).

Oil spill infrared sensing uses thermal infrared of wavelengths of 8–14 μm. Research on the 8–14 μm infrared shows that there are no spectral differences with thickness or other conditions [15]. Nighttime IR camera tests of IR cameras indicate that there is occasional oil detection (oil appears cool on a less-cool ocean); however, the definition is not as clear as in daytime [16]. Daytime images of oil using visible and infrared are shown in Figure 3 and Figure 4.

Infrared oil detection is not interference-free, because natural objects may appear like oil, such as seaweeds, sediment, organic matter, shoreline, and oceanic fronts. On the other hand, infrared sensors are cheap and are remote sensing tools that are readily available to the oil spill response worker. IR is not capable of slick thickness measurement.

### 2.5. Near-Infrared (NIR)

Near infrared (NIR) bands with wavelengths of 0.75–1.4 μm, have only a short time record in oil spills. NIR bands on the MODIS and MERIS satellites and the airborne AVIRIS were introduced and with it some research begun on oil spill applications. Oil spill remote sensing from these platforms was carried out during the Deepwater Horizon spill [17,18]. Two groups of researchers employed NIR to estimate oil thickness using the fact that reflection in the NIR varies somewhat with oil thickness [19,20,21]. Sun et al. [20] studied AVIRIS NIR data from the Deepwater Horizon spill to define the geometrics of slicks. An important finding of this study is that a minimal resolution is needed to display oil slicks accurately. It was found that, when 50% oil sheen fractional coverage was required, a 30 m resolution sensor was required.

The near-IR has also been used for other mapping purposes. One group delineated residual marsh oiling in Barataria Bay, Louisiana, after the Deepwater Horizon oil spill by using AVIRIS data [22,23]. Comparing the accuracy of the interpreted AVIRIS data over Barataria Bay resulted in an 87.5–93.3% accuracy compared to ground truth data. Shoreline oiling was found to extend 10.5 m into the marsh and showed a reduced oiling with increasing separation from the shoreline. Others have found some spectral reflectance spectra due to an emulsion as opposed to un-emulsified oil [24]. Further research on the use of near-IR is needed to extend its application to oil spills.

### 2.6. Ultraviolet (UV) 

Oil exhibits extensive sunlight reflectance in the UV. Ultraviolet sensors have been used to map oil sheens because sheens exhibit high UV reflectivity at low thicknesses (<0.1 μm) [3]. Superimposed UV and IR images sometimes historically were employed to yield a relative oil thickness map. This is currently not used, because the resulting thicknesses are not useful for spill cleanup techniques. Thickness measurements of 0.5–10 mm are required for oil spill cleanup work, these thicknesses are about three orders-of-magnitude greater than those estimated using IR [3], and that indicated by UV are much less. Ultraviolet images are subject to many oil look-alikes including: wind slicks, sun glints, and biogenic material [24]. Less UV use is being made for oil spills in today’s remote sensing because of the low relevance of thin slicks to oil spill cleanup.

### 2.7. Satellites Operating in the Optical Region

The application of visual satellite images for oil spills has been undertaken over the past few decades. Historically, there have only been a handful of satellites, all having low pass rates of as little as once every 26 days, so success was only on cloudless days when there was an overpass [3]. The primary problems were the timing of satellite overpasses and the absence of clouds [3]. The probability of the overpass and the clear skies occurring at exactly the same time gave a low chance of oil detection. This fact is illustrated during the Exxon Valdez spill, which covered large areas of sea for more than a month. There was a single cloudless day that corresponded with a satellite overpass, and that was on 7 April 1989 [3]. The other historical issue was the availability of algorithms to visualize the oil rather than the background. For the Exxon Valdez spill, experts required two months to produce a good image of the oil slick, although the slick coordinates were exactly known. At present, we now have many visual satellites and readily available algorithms to process the data.

QuickBird, WorldView I and II, new visual imaging satellites, now provide frequent coverage of the planet. Multi-spectral satellites such as MODIS and MERIS now provide various wavelength data for earth observation. Oil detection in the visible wavelength region depends on weather conditions, oil types, and view angles [25]. The barriers to visual imagery are cloud cover and sun glint. Sun glint is oftentimes severe and sometimes confounds an entire scene; however, a number of experts have made advances to remove sun glitter [26]. During the Deepwater Horizon spill, several visual images were employed [4]. Examples of this are shown in Figure 1 and Figure 2. Some researchers have employed MODIS data to characterize oil spills [26,27]. A multi-spectral image was derived from MODIS and corrected by a supervised classification system to enhance and characterize oil on water.

Satellite IR data was employed to delineate Kuwait land oil pollution [28]. Hydrocarbon-contaminated areas exhibited a thermal difference as great as 10 °C, compared to the nearby uncontaminated land. Ground-truth data were an essential part of the analysis process. Groups have used IR data from the AVHRR satellite to map oil spills [29,30]. The techniques typically employ the IR contrast difference between un-oiled and oiled water. Another group used MODIS SST (sea surface temperature) data to detect oil spills [3].

### 2.8. Image Processing

All visual imaging has been improved by the use of advanced image processing systems, especially satellite images have benefitted from this processing [31,32]. The algorithms can now automatically process images to correct them for aberrations, color, light levels, and most importantly, correspondence to GIS requirements. This has also reduced the delivery time of images from satellites, making these images useful to oil spill responders. Further, processing of optical images has enabled the fusion of different types of sensor outputs to produce new images [32].

### 2.9. Passive Microwave Sensors

Passive microwave sensors measure space-source microwave radiation as reflected from the sea [3]. Passive microwave radiometers detect the microwave emissivity difference between water and oil. The microwave signal detected also changes with oil thickness, so radiometry can be used to gauge oil slick thickness. However, there are interferences, and signal differentials may be poor. The microwave radiometer suffers from low spatial resolution. Spatial resolution is typically in tens of metres. Currently, microwave is not being used for slick imaging. Research emphasis is now on employing multiple microwave wavelengths to gauge slick thickness. Slick thickness measurements will be covered later in this paper.

## 3. Use of Active Sensors for Oil Spill Detection and Mapping

### 3.1. Laser Fluorosensors

**Figure 5:**
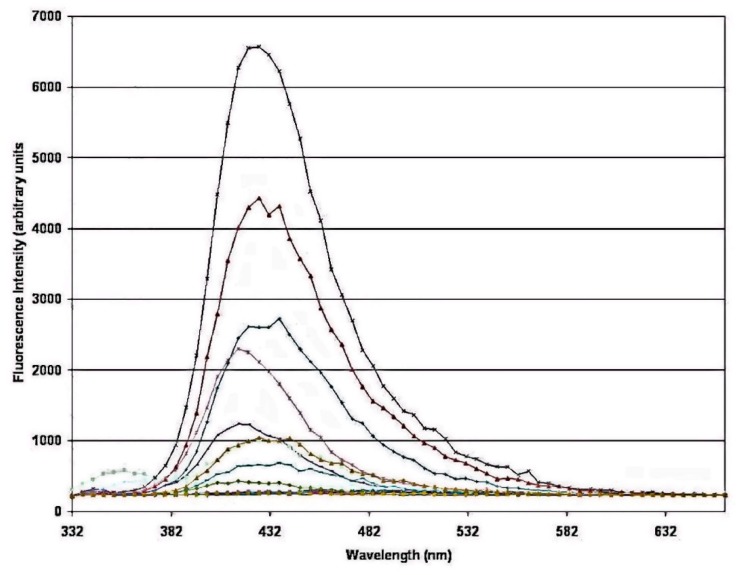
Fluorescence spectra of several light crude oils after activation with a 308 nm laser (Environment Canada). Spectra were obtained in a laboratory.

Laser fluorosensors use the phenomenon that oil aromatic compounds interact with ultraviolet light, absorb the light energy, and release the extra energy as visible light [33]. The absorption and emission wavelengths are unique to oil. Other substances in water, such as chlorophyll, fluoresce at distinctive wavelengths thus giving oil a unique signature. Various types of oil have distinct fluorescent intensities and spectral properties, resulting in the ability to differentiate different oil classes. The laser fluorosensor is best at discriminating between light, medium, and heavy oil types. Laser fluorosensors employ a UV laser operating between 308 and 355 nm [33]. UV lasers in the 300–355 nm region, such as the XeCl excimer laser (308 nm), the nitrogen laser (337 nm), the XeF excimer laser (351 nm), and the frequency-tripled Nd:YAG laser (355 nm), are available off-the-shelf. With an excimer laser excitation at 308 nm organic matter fluoresces at 420 nm. This is called “Gelbstoff” or yellow matter, which can be compensated for in fluorosensor output. Chlorophyll fluoresces at 685 nm. Crude oil fluoresces from 400 to 650 nm with the 308 nm excitation (Figure 5).

Some fluorosensors have detector activation at the precise time that the light returns from the target surface. This technique is called “gating”. This gating technique magnifies differentiation of the target oil from other possible interferences. Some fluorosensors can also gate their detectors to target areas below the sea surface. This enables detection in the water column. Work has been carried out with Orimulsion, a denser-than-water oil product [33]. This study was conducted using a gated fluorosensor that measured signal returns under the target sea surface. In this study, oil detection was possible as far as 2 m and easily at 1 m below a water surface. Laser fluorosensors are sampling instruments. The surface sampling rate is controlled by a laser repetition rate and the aircraft ground speed. At aircraft speeds of 120 knots and at a laser repetition rate of 100 Hz, a fluorescent sample is taken about every 60 cm along the flight path.

Raman scattering occurs when energy is exchanged between incident laser light and water [33]. Water molecules absorb UV light as rotational–vibrational energy and emit this energy in the form of light at a wavelength that is a difference between the incident radiation and the vibration–rotational energy of the molecule. The water Raman signal appears at 344 nm when the incident wavelength is 308 nm. The water Raman wavelength is an advantageous tool to maintain fluorosensor wavelength calibration.

**Figure 6:**
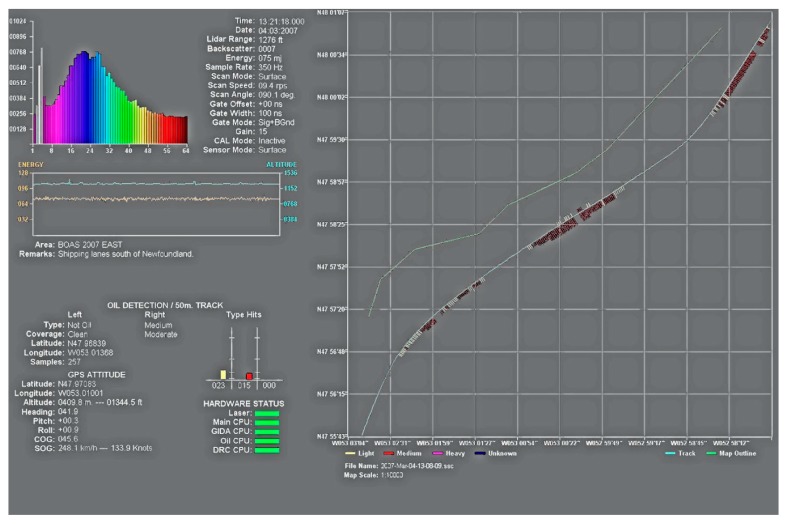
Operator’s monitor of the SLEAF system, showing sensor parameters and output map display (Environment Canada).

Laser fluorosensors are very useful oil spill sensors as they contribute a unique method of discriminating between oiled and unoiled seaweeds as well as detecting oil on shorelines. Tests on oiled shorelines demonstrated that laser fluorosensors can be very helpful [33]. Laser fluorosensors have a good track record in use and now are specified as essential in remote sensing packages. Laser fluorosensors provide a form of chemical analysis information to the user. The typical fluorosensor yields a plethora of user information as illustrated in Figure 6. Commercial instruments are now available and development of the technology continues.

### 3.2. Radar

Sea capillary waves reflect microwave (radar) signals, yielding an illuminated image called “sea clutter”. Oil on the sea attenuates capillary waves, and the sea clutter from radar imagery. Oil is then shown as a “dark” spot or a region of sea clutter absence [34]. Many substances also attenuate capillary waves. There are many slick look-alikes, such as fresh water slicks, calm areas, wave shadows behind structures or topographical features, shallow seaweed beds, biogenic oils, and sea-life sperm [3]. In certain areas, slick look-alikes could number in the hundreds. Liu et al. revealed that slick look-alikes in the studied synthetic aperture radar (SAR) imagery were as high as 20% even after extensive processing [35]. Even with these many look-alikes, radar is essential for oil spill remote sensing, as radar is uniquely capable of operating at night and with clouds or fog.

There are two configurations of radar: SAR and side-looking airborne radar (SLAR). SLAR is cheaper and uses a horizontal antenna to produce imagery along the flight path. Synthetic aperture radar uses the aircraft forward motion of the aircraft to attain spatial resolution. SAR resolution is not dependent on range, but uses extensive electronic processing to produce high resolution images. SAR has larger range and resolution than SLAR. Comparative testing indicates that SAR has several advantages and is used in all radar satellites [4]. SLAR is mostly used for airborne oil spill remote sensing, as it is cheaper.

Studies on oil spill remote sensing indicate that X-band radar provides better data than L- or C-band radar [34]. The lengthy availability of oil during the Deepwater Horizon spill provided several researchers with the opportunity to study radar remote sensing as well as band relationships [36]. Overall, X-band is superior to other bands, but C-band radar and even L-band radar, to a degree, can provide useful oil spill data [34]. The contrast between oil and water is highest in X-band, moderate in C-band, and lowest in L-band [34].

Signal polarizations using vertical (V) and horizontal (H) electromagnetic wave propagation can be used to provide further information [37]. Transmission and reception using the same polarization is denoted by the repetition of the letters, i.e., VV or HH (transmission polarity then reception polarization). Four poles are available: HH, VV, HV, and VH and if all four poles are used, the polarization is called quadrupole. Vertical antenna transmission and reception polarizations (VV) produces better imagery using airborne radar. A new form of polarimetry is compact SAR, in which the system uses a mixed polarization where the transmitter polarization is either circular or orientated at 45 degrees and the receivers are aligned horizontally and vertically [38]. Compact SAR a form of coherent dual-polarity has advantages over single-pole work and has been used on oil spills. Polarimetric SAR yields information to aid in the discrimination between slicks and look-alikes [39,40].

Sea state limits radar ability to image oil. Low sea states do not create sufficient sea clutter to contrast with the oil that has damped sea clutter. High seas scatter radar to the extent that detection is not possible inside the wave troughs. Minimum wind speeds of 1.5 m/s (~3 knots) are required for detectability, and a maximum wind speed of 6–10 m/s (up to 20 knots) will again hamper radar detection [4].

Radar also detects dampening agents that appear to be like oil. New means of data processing has attempted to eliminate these look-alikes from imagery and automate the process of slick detection [41,42].

**Figure 7:**
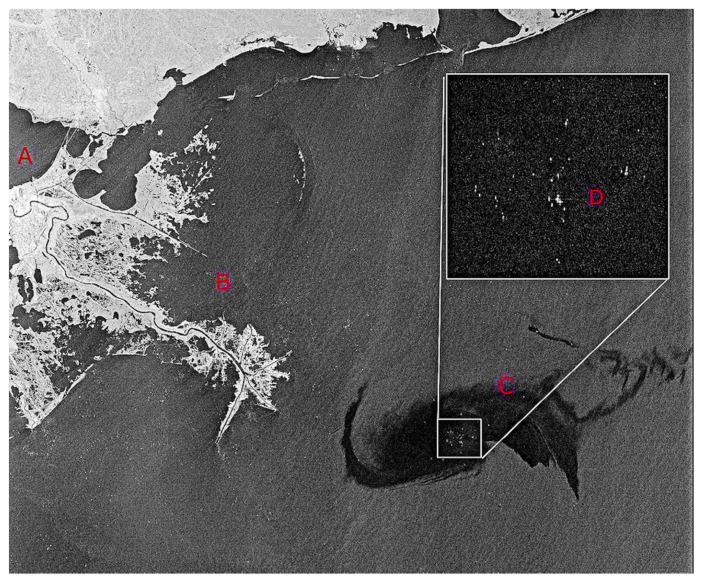
Radarsat imagery of the Deepwater Horizon spill. The square shows an expanded view of the center of the spill area and the bright dots are ships working to control the spill.

Overall, radar is a very good sensor for large-area, night-time, and foul weather detection work. Radar imagery is susceptible to look-alikes and has wind speed limitations (1.5–10 m/s). Radar is now the standard for mapping oil offshore. Many satellite options with extensive coverage are now available to the user. Figure 7 shows radar imagery that was used for the Deepwater Horizon spill.

### 3.3. Satellite Radar Systems

There are now several radar satellites in orbit providing users with a choice of coverage and polarizations. Table 1 lists present and proposed radar satellites. Most radar satellites provide a selection of resolutions and polarizations. While many satellites have a lifetime of 5 years, some satellites have functioned for 17 years.

**Figure 8:**
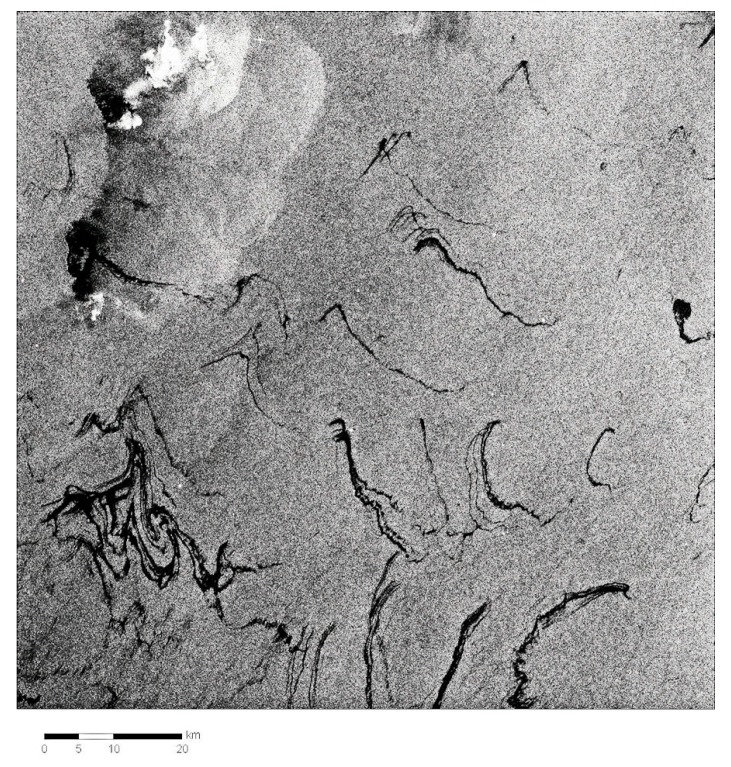
RADARSAT-2 view of leaking oil platforms in the Caspian Sea (Image from Macdonald-Detwiler).

**Figure 9:**
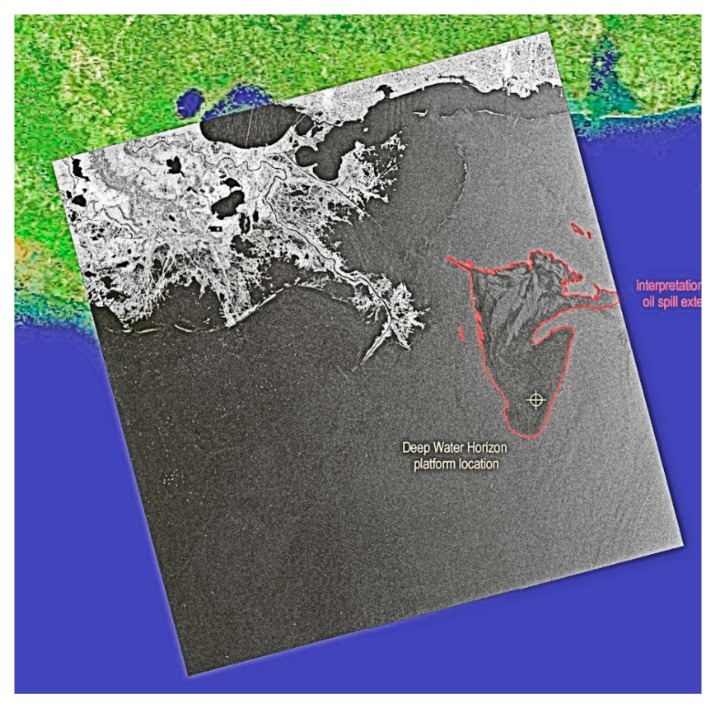
RADARSAT-2 image of the Gulf oil spill. The red outline shows the spill. The shoreline is shown as white and the colored portions derive from a visible satellite image (Image from Canadian Space Agency website http://www.asc-csa.gc.ca/images).

**Figure 10:**
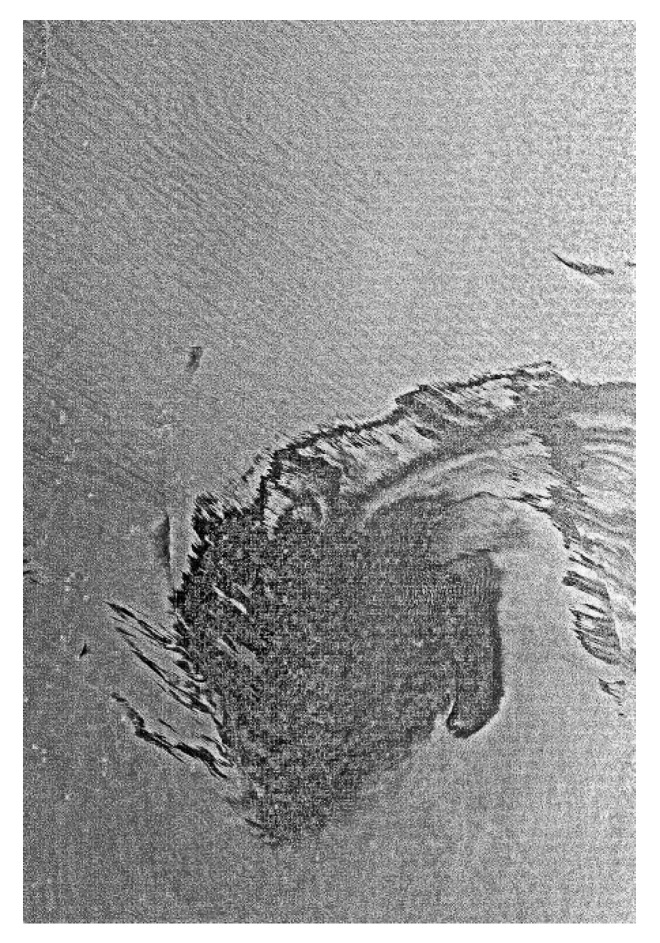
RADARSAT-2 view of a small portion of the Gulf oil spill. This is detail from another wide-field image to show the detail that radar is capable of. The white dots are ships working on well control. (Image from Canadian Space Agency website http://www.asc-csa.gc.ca/images).

The extensive 2010 Gulf of Mexico oil spill provided opportunity for testing and use of satellite radar data. Some of this use and other worldwide uses are shown in Figure 7, Figure 8, Figure 9 and Figure 10.

Delivery time of radar imagery is a consideration. In the past, times from the tasking of the satellite to image delivery were as long as 12 h, currently, times of 4 h are possible. A further consideration is overpass time. Many satellites will cover an area once per day. This has been improved by use of satellite constellations [43]. The larger number of SAR satellites and satellite constellations such as Cosmo (Constellation of Small Satellites for Mediterranean basin Observation) will give revisit times of a few hours compared to the present one-day. 

### 3.4. Radar Image Processing

There are many spill look-alikes which interfere with radar imagery. Many studies have been devoted to removing the look-alikes from radar imagery [44]. Further, there are other aberrations to oil spill mapping by radar. Extensive efforts have been devoted to automating this interference-elimination process and radar data processing in general. The data processing steps generally follow a procedure which will be outlined here. An important first process is to assess the particular image for quality [45]. If the image meets certain quality requirements, then it will be advanced for further processing. The next step then becomes removal of noise and speckle. Noise is contained in many radar images. Noise can complicate subsequent analysis and therefore should be reduced [46]. Speckle is a special kind of granular noise. Speckle noise results from constructive and destructive interference of the signal shown as bright and dark dots in the image. The presence of speckle may negate further processing. There are several methods to remove speckle, one of which is to average several images of the same area over time [47]. 

The next step in radar image processing is the removal of wind fields and fixed geographic features. Radar detection of oil is difficult or impossible in low or high wind areas. It is then imperative to compare radar imagery to the wind field for that area [48]. One method of dealing with winds is to use a wind map of same scale as the radar image for direct comparison.

Land and near-shore areas constitute an interference to radar detection of oil spills. Processing of radar imagery alongside a graphical information systems (GIS) can eliminate these no-oil-detect areas such as areas close to shore [46]. Similarly known weed beds or other interferences can be blanked out.

The third step in radar processing is to locate the oil “dark” spots in the image with the highest accuracy. Edge detection is an imagery processing method which find the distinct boundary between the target feature and background [49]. Several mathematical algorithms have been developed for edge detection in radar images. Texture analysis is a technique to distinguish the area surface characteristic of imagery and has been applied to radar imagery of oil [50]. Oil generally shows a consistent texture, whereas sea has a rougher texture. Another way to approach the detection of the black oil areas is to use shape analysis. Oil shows different shapes compared to look-alikes. These differences have been used to differentiate oil from look-alikes [51,52].

Intelligent systems are often used to assist in image processing, sometimes including the above steps. The basic procedure involves manual input into a system that follows the steps and then uses this “learning” to perform the process by computerized procedures [53,54,55]. Development of these techniques has now lead to several systems that are automatic or semi-automatic and can interpret a radar image for oil slicks in a matter of minutes. Algorithms for radar interpretation are also available to subscribed users on the internet. This vast improvement has occurred in the past 10 years.

### 3.5. Ship-Borne Radar Oil Spill Detection 

Ship-borne radar was in existence for many years and only recently has been modified to include oil spill detection and mapping. Ship-borne radar has a range of 8–30 km, as a function of antenna height [56]. Ship-borne radars are becoming a dependable method of detecting and mapping slicks close to the recovery vessels and fulfilling a tactical need for imagery directly on recovery ships.

## 4. Slick Thickness Measurements 

Slick thickness is a strong need for various applications ranging from countermeasures effectiveness assessment to legal purposes for prosecution [57]. Slick thickness measurement is problematic because there are very few operational methods. Second, the visual color methods currently tried may be incorrect. There are a few simple techniques to estimate slick thickness in the range 0.1–5 μm (0.0001–0.0005 mm), but these do not relate to most oil spill needs. Spill work requires thickness measurements greater than 0.5 mm up to about 10 mm [57].

### 4.1. Passive Microwave

Passive microwave has long been used as an indicator of oil slick thickness. The microwave brightness of the slick varies with the thickness, but in a cyclical fashion. This means that a particular microwave brightness implies one of several thicknesses [57]. One method to overcome this is to use multiple wave lengths. This will reduce ambiguity to a useable degree. One should note, however, that the microwave brightness is also influenced by a number of other factors such as weather and sea conditions and type of oil. Another important factor one should note, is that in practice, the microwave response requires calibration to determine the response factors. The best way to do this is to use simulants such as plastic sheets and integrate volume back to the amount released. Once one instrument is “calibrated” in this manner, it is useable for field work.

In the 1970s, Hollinger and Mennella studied a series of small test slicks off the coast of New Jersey [58]. Using a two-frequency microwave receiver, they plotted the thickness of the slicks. They found that 90% of this fresh slick volume was contained in 10% of the area and vice versa, this number was subsequently found to vary from slick to slick and could not be used as a “rule of thumb” [57]. Microwave thickness measurement technology continued to evolve for the next 15 years. Skou et al. reviewed microwave sensing extensively and carried out experiments to measure oil thicknesses using a three-frequency radiometer (5, 17, and 34 GHz) [59]. It was shown that an accurate thickness profile could be drawn, given major adjustments. 

This highlighted the problem with early devices; the early selection of frequencies and calibration methodology did not allow for the measurement of slicks thicker than about 1 mm.

**Figure 11:**
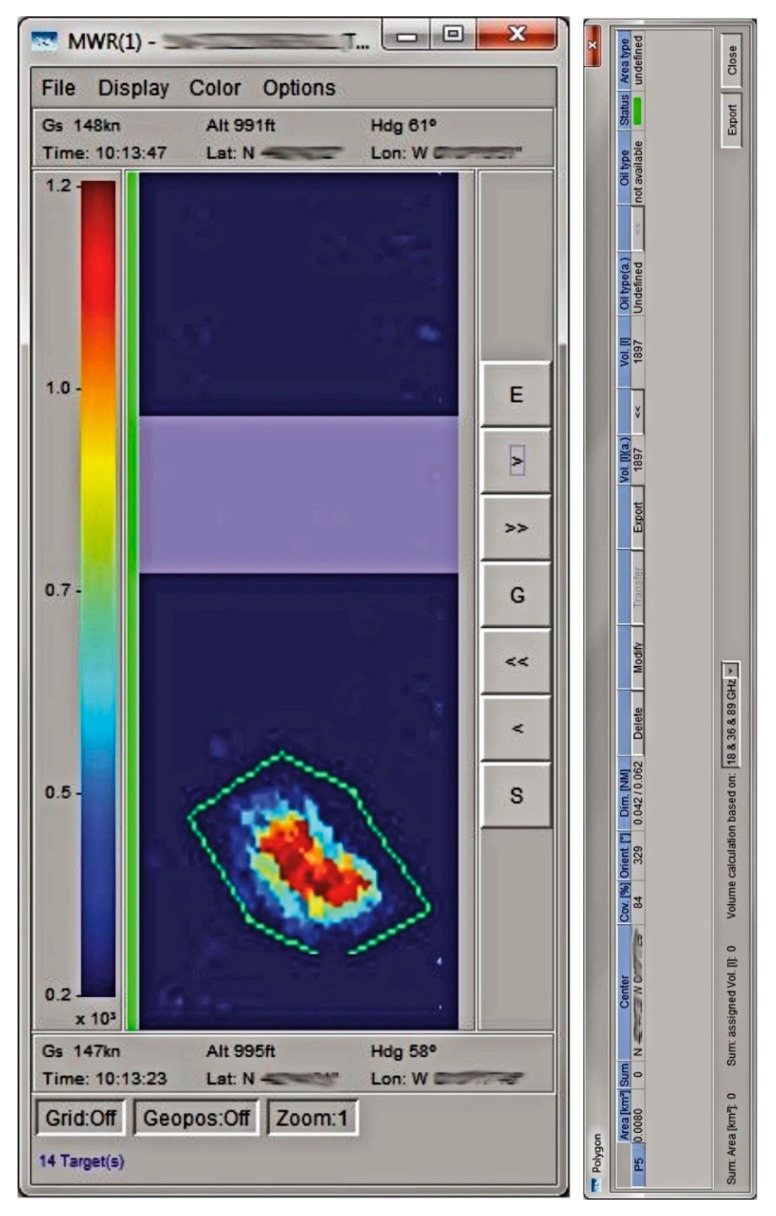
A slick thickness profile from a passive microwave sensor (photo from Nils Robbe, Optimare). The colors represent thicknesses as shown on left hand scale (thickness ranges from 0.2 to 1.2 mm in this case). This image was taken directly from an operating aircraft display. The information available on the display includes location, area of slick (here 0.008 km^2^), and oil volume (here 1897 L). The microwave instrument was a 3-band passive microwave with receivers at 18, 36, and 89 GHz.

In the next decade, microwave sensor technology developed to the point of commercialization. The only instruments currently available for measuring slick thickness are the Optimare three- to five-channel microwave instruments [60]. These instruments have the capability of measuring from 0.05 to 3 mm. A sample slick output from this sensor is shown in Figure 11. There is little public data available, as most of the existing measurements are used for legal prosecution purposes, but these instruments are extensively used. Questions remain, however, on the applicability of this technology to oils containing water, such as emulsions.

### 4.2. Visual Appearance

Historically, several workers tried to correlate the thickness of oil to its visual appearance and color. Further study indicates that this technique is limited to sheens and rainbow-colored slicks [57]. Physically based thicknesses of about 0.7–2.5 mm appear as rainbow colors as a consequence of multiple constructive and destructive light interferences. Sheen is the result of sunlight reflection from thinner oil layers. Some workers note that visual appearance and colors should be limited to thin oil or sheen, and thick oil categories [61].

### 4.3. Infrared Brightness

Infrared imagery may appear to show thickness gradations as a result of different surface characteristics, but extensive testing by several researchers showed that the brightness of the infrared is invariant with slick thickness [14,57]. Thus, infrared sensing does not yield slick thickness measurements.

### 4.4. Acoustic Travel Time 

The acoustic travel time of oil is an indication of thickness as the acoustic velocities through most oils is similar [62]. One method of creating the acoustic pulse is to use a CO_2_ laser. The rapid heating in the oil caused by the absorption of the infrared signal sets up an acoustic signal in the oil. A portion of the acoustic wave in the oil is reflected by the water/oil interface and travels back to the oil surface. The reflected acoustic wave on the surface can be measured by another laser. The acoustic signal appears in the reflected laser signal by Doppler shift, and the audio can be demodulated from the laser signal. The travel time of the acoustic pulse is directly related to the thickness. A prototype instrument using this principle has been built and tested over various pool thicknesses and was able to measure these thicknesses.

## 5. Detection of Oil on the Sea Bottom

Sunken oil is a problem [63]. Several methods, as described below, have been used to remotely detect sunken oil.

### 5.1. Ultrasonics

Oil has a different acoustic profile than soft sea bottoms or hard sea bottoms. This small difference enables limited detection of oil on the sea floor using ultrasonics [64]. In each locale, ground-truthing is required to ascertain the characteristics of the sea floor versus that of the oil.

### 5.2. Laser Fluorosensors

Laser fluorosensors can detect the aromatic compounds in oil. Laser fluorosensors were tested for their ability to detect oil in the water column. This was shown to be viable to a depth not exceeding about 2 m [33]. Although suggested several times, laser fluorosensors operating on the surface have not been tested for their capability of detecting oil on the sea floor. Sub-sea fluorosensors have been successful however in limited testing.

### 5.3. Cameras

Cameras operating in the visual spectrum have been used for mapping oil on the bottom of a water body [63]. Extensive ground-truthing may be required if the oil does not contrast with the bottom material.

### 5.4. Chemical Analysis

Hydrocarbons in the water column and possibly over sunken oil can sometimes be detected using chemical analysis such as mass spectrometry or fluorometer [65].

## 6. Concluding Remarks

Recent years have shown some progress in oil spill sensor development. Sensor manufacturers see oil spill applications as a small sector, so limited development has occurred. Cameras have improved very much and could be enhanced by adding specific filters or polarizing lenses. The situation is similar for IR cameras. The claimed new results for near IR have not been exploited nor substantiated by practical application. Satellite application of optical techniques has been limited by cloud cover and lack of contrast, despite the increased availability of new platforms.

There are now some commercial laser fluorosensors available and these are generally being applied to surveillance of illegal discharges. Satellite radar is now dominating offshore surveillance. Several satellites are now providing data in several different modes. Radar processing is now very advanced. The strategic advantages of radar satellite data are difficult to match. Ship-mounted radars has provided very good tactical support for oil spill recovery vessels.

There is little technology improvement for near-shore and land spills in comparison to spills on the sea. There have been no improvements in two decades. A similar situation exists for sub-surface spills and for oil in ice situations. The passive micro-wave technique for thickness measurement remains the only commercial option at this time. The sensor requires more utilization to develop confidence with different oil types and states.

The automated airborne drone may be the tactical and operational support platform for the future. At this time, drones are equipped with visible and infrared cameras.

## Figures and Tables

**Table 1:** Satellite-borne synthetic aperture radar (SAR) sensors—current and future.

Satellite	Launch Date	Owner/Operator	Band	Polarization
ERS-1	1991 (end 2000)	European Space Agency	C	
ERS-2	1995 (end 2011)	European Space Agency	C	VV
RADARSAT-1	1995 (end 2013)	Canadian Space Agency	C	HH
RADARSAT-2	2007	Canadian Space Agency	C	
ENVISAT (ASAR)	2002 (end 2012)	European Space Agency	C	HH, VV, Cross pol
ALOS (PALSAR)	2006 (end 2011)	Japan Aerospace Exploration Agency	L	
TerraSAR-X	2007	German Aerospace Centre	X	
Tandem -X	2010	German Aerospace Centre	X	
Cosmo Skymed-1/2	2007, 2010	Italian Space Agency	X	
TecSAR	2008	Israel Aerospace Industries	X	
Kompsat-5	2013	Korean Space Agency	X	
Sentinel-1	2013	European Space Agency	C	
RADARSAT-Constellation	2018	Canadian Space Agency	C	
(3-satellites)				
